# Postural control processes during standing and step initiation in autism spectrum disorder

**DOI:** 10.1186/s11689-019-9305-x

**Published:** 2020-01-06

**Authors:** Erin K. Bojanek, Zheng Wang, Stormi P. White, Matthew W. Mosconi

**Affiliations:** 1 Life Span Institute and Clinical Child Psychology Program, University of Kansas, 1000 Sunnyside Ave., Lawrence, KS 66045 USA; 2 Kansas Center for Autism Research and Training (K-CART), University of Kansas Medical Center, Overland Park, KS 66213 USA; 3 Department of Occupational Therapy, University of Florida, 1225 Center Drive PO Box 100164, Gainesville, FL 32611 USA; 4 Marcus Autism Center, Department of Pediatrics, Emory University School of Medicine, 1920 Briarcliff Road, Atlanta, GA 30329-4010 USA

**Keywords:** Autism Spectrum disorder, Postural control, Mutual information, Stepping, Anticipatory postural adjustments

## Abstract

**Background:**

Individuals with autism spectrum disorder (ASD) show a reduced ability to maintain postural stability, though motor control mechanisms contributing to these issues and the extent to which they are associated with other gross motor activities (e.g., stepping) are not yet known.

**Methods:**

Seventeen individuals with ASD and 20 typically developing (TD) controls (ages 6–19 years) completed three tests of postural control during standing. During the neutral stance, individuals stood with their feet shoulder width apart. During the Romberg one stance, they stood with feet close together. During the circular sway, participants stood with feet shoulder width apart and swayed in a circular motion. The standard deviation (SD) of their center of pressure (COP) in the mediolateral (ML) and anteroposterior (AP) directions and the COP trajectory length were examined for each stance. We also assessed mutual information (MI), or the shared dependencies between COP in the ML and AP directions. Participants also completed a stepping task in which they stepped forward from one force platform to an adjacent platform. The amplitude and duration of anticipatory postural adjustments (APAs) were examined, as were the maximum lateral sway, duration, and velocity of COP adjustments following the initial step. We examined stepping variables using separate one-way ANCOVAs with height as a covariate. The relationships between postural control and stepping measures and ASD symptom severity were assessed using Spearman correlations with scores on the Autism Diagnostic Observation Schedule–Second Edition (ADOS-2) and the Autism Diagnostic Interview-Revised (ADI-R).

**Results:**

Individuals with ASD showed increased COP trajectory length across stance conditions (*p* = 0.05) and reduced MI during circular sway relative to TD controls (*p* = 0.02). During stepping, groups did not differ on APA amplitude (*p* = 0.97) or duration (*p* = 0.41), but during their initial step, individuals with ASD showed reduced ML sway (*p* = 0.06), reduced body transfer duration (*p* < 0.01), and increased body transfer velocity (*p* = 0.02) compared to controls. Greater neutral stance COP_ML_ variability (*r* = 0.55, *p* = 0.02) and decreased lateral sway (*r* = − 0.55, *p* = 0.02) when stepping were associated with more severe restricted and repetitive behaviors in participants with ASD.

**Conclusions:**

We found that individuals with ASD showed reduced MI during circular sway suggesting a reduced ability to effectively coordinate joint movements during dynamic postural adjustments. Additionally, individuals with ASD showed reduced lateral sway when stepping indicating that motor rigidity may interfere with balance and gait. Postural control and stepping deficits were related to repetitive behaviors in individuals with ASD indicating that motor rigidity and key clinical issues in ASD may represent overlapping pathological processes.

## Background

Sensorimotor deficits, including reduced postural control [1–3], poor upper and lower limb coordination during reaching [4] and walking [5, 6], and reduced anticipatory control of motor behaviors [7, 8], frequently are seen in individuals with autism spectrum disorder (ASD) and are considered associated features supporting a diagnosis [9]. Multiple motor control mechanisms used to maintain stability and initiate movements have been implicated in ASD, including sensory feedback, motor coordination, and feedforward processes. In order to ensure precision and minimize variability during ongoing motor behaviors (e.g., maintaining a steady posture during standing), sensory feedback inputs are used to continuously adjust behavioral output [10–14]. Control of continuous motor behaviors also involves the coordination of distinct joint movements [10]. Coordinating distinct joint movements allows individuals to both complete complex movements (e.g., reaching for and grasping an object) and skillfully adjust their balance to maintain control (e.g., adjusting posture to maintain balance while initiating a step). Feedforward motor processes also play a prominent role in ensuring control of continuous motor behavior as they are involved in planning and executing initial or rapid movements made prior to sensory feedback being available [15]. The present study aimed to develop a more mechanistic understanding of postural control in individuals with ASD by investigating reactive adjustments of posture in response to sensory feedback, the coordination of distinct postural control processes, and feedforward processes involved in anticipatory postural adjustments made prior to initiating stepping. Based on evidence that sensory feedback, coordination, and feedforward processes involve distinct brain networks, determining the extent to which these motor control mechanisms are impaired in ASD during postural activities may provide key insights into neural processes associated with the disorder.

Studies of postural control in ASD have implicated sensory feedback processes. Individuals with ASD are less stable than typically developing (TD) controls during standing [1, 2, 16–18], and they show more severe postural control deficits, including increased postural sway, when sensory feedback information is occluded or removed [1, 3, 17, 19]. These findings suggest that individuals with ASD may place greater demands than controls on feedback processes in order to maintain stability. Individuals with ASD also show more severe postural control deficits during dynamic postures. For example, Wang et al. (2016) found that individuals with ASD demonstrated increased center of pressure (COP) variability during a dynamic stance condition in which they intentionally swayed along the mediolateral (ML) or anteroposterior (AP) axis. Elevations in COP variability in ASD were more severe during the dynamic compared to a static standing condition, suggesting that individuals with ASD show greater levels of impairment during conditions in which demands on feedback motor control processes are increased [18]. While few studies have examined postural control during dynamic conditions, control during dynamic postural adjustments more closely relates to activities of daily living than static stances and therefore may be informative for determining more clinically relevant postural issues. The current study will address key gaps in our understanding of postural control processes in ASD by examining feedback mechanisms that support postural stability across both static and dynamic stance conditions that more closely relate to tasks of daily living than previously studied postural tests.

Coordination of joint movements is necessary to maintain postural stability. When standing still, processes that control ML sway, including abduction and adduction of hip joints, and AP sway, including dorsi and plantar flexion of ankle joints, are coordinated to simultaneously correct sway in multiple directions [18, 20, 21]. During static stance in healthy individuals, these distinct processes show moderate cross-talk, or mutual information (MI), which helps modulate sway in all directions. In healthy individuals, elevated levels of MI allows for the coordination of distinct joints during more challenging stance conditions (e.g., when making rapidly shifting movements or when visual information is removed) [22] and increased MI during these conditions appears to be associated with decreased variability. In contrast, reduced MI is optimal during more directional sway suggesting that distinct ankle and hip processes operate more independently to ensure that sway is directional. Wang et al. (2016) examined joint coordination in children with ASD to determine the extent to which postural control processes that control AP sway, operated independently or in concert with processes that control ML sway. When engaging in single direction sway, individuals with ASD showed a reduced ability to decouple hip and ankle joint control processes as reflected by increased MI relative to controls [18]. These findings indicate that individuals with ASD show deficits in adaptively modulating the degree of coordination among distinct postural control processes during dynamic postural conditions. By determining the extent to which individuals with ASD are able to flexibly coordinate different joint processes, tests of naturalistic dynamic postures may provide new insights into motor control mechanisms contributing to postural deficits in ASD.

Feedforward, or predictive, postural control processes are critical for planning movements or shifting bodyweight in order to maintain postural stability prior to the onset of a goal-directed movement, such as walking. Individuals with ASD show a reduced ability to predictively modulate neuromuscular movements, as demonstrated by reduced amplitude of anticipatory postural adjustments (APAs) made prior to predictable upper body movements, and reduced cortical activity prior to the onset of APAs [7, 8]. Reduced APA modulation may contribute to previously reported postural and gait alterations in ASD [5], though the distinct processes that underpin gait abnormalities in ASD have not been systematically assessed. Stepping involves three distinct phases: (1) the anticipatory phase including APAs made prior to the initial step, (2) the body weight transportation phase which includes shifts in individuals’ COP during the interval between the heel strike of the lead foot and the moment the toe of the back foot lifts off of the ground, and (3) the follow through phase comprised of the interval between the back toe lifting off of the ground to the point in time when the individual resumes standing still with both feet together. While the initial anticipatory phase is dominated by feedforward processes, the transportation and follow through phases involve dynamic integration of feedforward and feedback processes in order to maintain fluid and stable movements. During stepping, individuals with ASD show reduced lateral sway when initiating a step [5], reduced stride length [23], and decreased peak plantar flexion and hip flexor movements associated with hypotonia [24]. In order to determine the extent to which feedforward postural control processes relate to feedback and coordination processing used during step initiation and other postural control conditions, we examined postural control during the anticipatory and body transfer phases of stepping in order to measure both feedforward and feedback mechanisms supporting step initiation.

The present study examined feedback, coordination, and feedforward processes of postural control across static and dynamic standing conditions and stepping in order to develop a more mechanistic understanding of postural impairments in individuals with ASD. There were three primary aims. First, we examined feedback mechanisms used to support postural stability. Based on prior findings that individuals with ASD show atypical processing of sensory feedback information and increased sway when standing still [3], we predicted that individuals with ASD would show increased COP variability and COP trajectory length across all stance conditions relative to controls. Further, we expected that the severity of postural control deficits in ASD would increase as greater demands were placed on feedback motor control processes, as in the circular sway condition. Second, we examined the coordination of distinct postural control processes used to support ML and AP adjustments in ASD across all standing conditions. These distinct processes were analyzed in order to determine the extent to which individuals with ASD are able to effectively coordinate their joint movements during standing conditions in which increased MI is advantageous in contrast to our prior study examining single direction sway when MI should be reduced [18]. We hypothesized that, compared to controls, individuals with ASD would show reduced MI during circular sway relative to controls. Finally, we examined stepping to understand feedforward and feedback mechanisms of postural control during walking. We hypothesized that APAs during the anticipatory phase would be smaller in amplitude and duration for individuals with ASD compared to controls. During the body weight transportation phase, we hypothesized that individuals with ASD would show reduced lateral sway suggesting greater instability when stepping. Based on previous findings showing that deficits in postural control are associated with more severe ASD symptoms [25, 26], we also investigated the extent to which our measures of postural control and stepping were associated with social-communication abnormalities and repetitive behaviors in ASD.

## Methods

### Participants

Seventeen individuals with ASD (ages 6–19 years) and 20 TD control individuals matched at the group level on age, sex, non-verbal IQ, and body mass index (BMI) completed tests of postural control and step initiation (Table 1). IQ was assessed using the Wechsler Abbreviated Scales of Intelligence [27], and all participants were required to have an IQ > 70. One TD control individual was unable to complete IQ testing due to scheduling difficulties, but no history of learning or developmental concerns were indicated on caregiver report, so this control participant was retained. Individuals with ASD were recruited through community advertisements and local clinics. For all ASD participants, a diagnosis of ASD was established using the Autism Diagnostic Inventory-Revised (ADI-R) [28], the Autism Diagnostic Observation Schedule–Second Edition (ADOS-2) [29], and expert clinical opinion based on DSM-5 criteria [9]. For ADOS-2 testing, one participant completed Module 2, 14 participants completed Module 3, and two participants completed Module 4. Three ASD participants’ parents were unable to complete the ADI-R, but these participants met ASD classification on the ADOS-2 and DSM-5 criteria for ASD. Potential participants were excluded if they had any known genetic condition associated with ASD.

**Table 1:** Demographic and clinical characteristics of participants with ASD and TD controls

	ASD (*n* = 17)	TD (*n* = 20)
Age (years)	13.67 (3.00)	12.48 (4.17)
Height (cm)	161.80 (15.88)*	149.46 (17.61)*
Weight (kg)	58.82 (15.41)*	45.81 (18.41)*
Leg Length (cm)	85.31 (9.13)	77.39 (15.34)
BMI	22.18 (4.04)	19.71 (4.13)
% Male^a^	88%	80%
FSIQ	97.76 (17.27)	108.47 (14.53)
PIQ	99.76 (16.41)	104.32 (11.57)
VIQ	96.18 (17.46)*	110.58 (15.34)*

*FSIQ* full-scale IQ, *PIQ* performance IQ, *VIQ* verbal IQ

*Note.* Data are reported as mean and standard deviation in parentheses

^a^% Male was compared across groups using a chi-square test

*p < 0.05

TD participants were recruited from the community and scored eight or lower on the Social Communication Questionnaire (SCQ) [30]. TD participants were excluded for current or past psychiatric or neurological disorders, family history of ASD in first- or second-degree relatives, or a history of developmental or learning disorders, psychosis, or obsessive-compulsive disorder in first-degree relatives based on a screening interview.

No participants were taking medications known to affect motor performance at the time of testing, including antipsychotics, stimulants, or anticonvulsants [31]. No participant had a history of head injury, birth asphyxia, or non-febrile seizure. Participants 18 years of age or older provided written consent, and minors provided assent in addition to written consent from their parent or legal guardian. All study procedures were approved by the local Institutional Review Board.

### Apparatus and procedures

Participants completed three postural control tasks using one AMTI (American Mechanical Technology, Inc., Watertown, MA) force platform (Model: AccuGait; size: 49.78 × 49.78 cm; sampling rate: 1000 Hz) and one stepping task using two adjacent AMTI force platforms. The four experimental conditions totaled 30–40 min. Participants rested for 30 s between each trial and for 1 min between conditions; additional breaks were given when necessary to ensure valid data acquisition. Three successful trials were completed for each of the four conditions. Prior to each condition, the experimenter modeled the task. Participants were given practice before the test to ensure they understood the instructions.

#### Static stances

Two static standing conditions were administered: neutral stance and Romberg one stance. During the neutral stance, participants stood as still as possible with their feet shoulder width apart and their arms resting at their side. In order to examine postural control during a more challenging condition that is commonly used to assess for cerebellar dysfunction, participants also completed a Romberg one stance [32] in which they stood with their feet side-by-side and arms at their sides.

#### Dynamic stance

During the test of circular sway, participants stood with their feet hip width apart and made a circle with their body at a natural speed. Data collection began 5 s after the participants started their circular sway.

Participants completed three 30-s trials for each static and dynamic condition (3 trials × 3 conditions = 9 trials). Tracings of participants’ feet were collected before the start of each postural stance and participants stood on the tracing and were instructed not to move their feet during testing. COP_AP_ and COP_ML_ variability, MI, and COP trajectory length were measured for each static stance condition.

#### Stepping task

Participants completed trials in which they took one step forward from one force platform to an adjacent force platform. During each trial, participants naturally stood on the posterior force plate for 3 to 5 s, received an auditory cue of either “right” or “left” prompting them to step with their right or left foot, and then stepped towards the anterior force platform at a comfortable speed and distance. In order to finish the trial, participants needed to have both feet resting on the anterior platform in their neutral stance. The direction of the auditory cue was randomized across trials, and the timing was randomly presented within 3 to 5 s after the experimenter confirmed that the participant was standing still on the first platform. Each trial was followed by 10 s of rest. Three successful trials were collected for each participant. COP data from each trial were used to calculate APA duration and amplitude as well as maximum lateral sway, body weight transfer duration, and body weight transfer velocity. The order of administration of static and dynamic standing conditions and the stepping task was counterbalanced across participants.

#### Data processing and analysis

All kinetic data (force and moment time series) were down sampled to 100 Hz and low pass filtered using a fourth-order double pass Butterworth filter with a cutoff frequency of 6 Hz in Matlab 2016b (MathWorks, Inc., Natick, MA). For the static and dynamic standing conditions, the first 5 s and the last 5 s of data were removed in order to eliminate extraneous movements made while participants started and ended the task. For the postural control conditions, the COP time series for the force platform was derived from force and moment data consistent with prior methods [33]. The COP_NET_ includes the COP time series in both AP (COP_AP-NET_) and ML (COP_ML-NET_) directions. The COP_NET_ time series were derived from COPs as well as the vertical ground reaction force [34, 35]. For the stepping test, COP time series were extracted for each force platform and derived in an identical manner as for the standing conditions. To assess participants’ postural stability during the static and dynamic standing trials, we measured the standard deviation (SD) of the COP time series in both the AP and ML directions as well as the resultant COP trajectory length, or the sum of distances between points on the COP path [33].

To examine postural coordination during static and dynamic standing conditions, we measured the amount of MI shared between the COP in ML and AP directions. COP_AP_ primarily is controlled by ankle dorsi/plantar flexion while COP_ML_ is more associated with movements of the hip abduction/adduction [18, 20, 21, 36]. MI is a measure of shared dependency between two time series, and the unit is bit [18, 37]. In this study, MI is used to quantify the amount of shared information between COP_AP_ and COP_ML_ during different postural control conditions. Higher MI suggests more shared information across the COP_AP_ and COP_ML_ time series while lower MI suggests independent movements in the AP and ML directions [18].

**Fig. 1:**
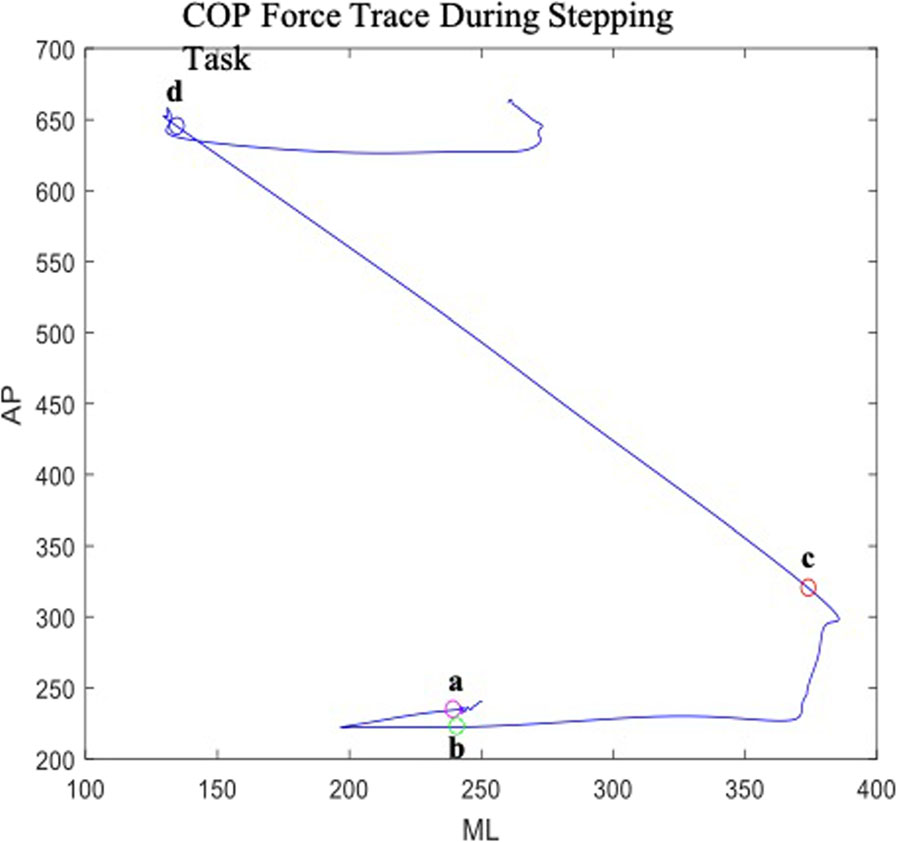
COP force trace during stepping task. **a** The onset of the anticipatory phase was defined as the first point where the COP in the ML direction was greater than two SDs from the baseline and remained for at least 50 ms. **b** The APA offset was defined as the point where the COP returned back to baseline. **c** The point where the stepping foot lands on the anterior force platform. **d** The point where the back foot leaves the posterior force platform

The stepping task was separated into anticipatory and body transportation phases. To examine the anticipatory phase, APA amplitude and duration were examined. The onset of the anticipatory phase was defined as the first point where the COP in the ML direction was greater than two SDs from the baseline stance and remained for at least 50 milliseconds (Fig. 1a). The APA offset was defined as the point where the COP returned back to baseline before beginning the step (Fig. 1b). The APA contains an ML shift towards the stepping leg before the shift towards the standing leg and the onset of the step. APA amplitude is the maximum range of motion in the ML direction throughout the APA phase. The APA duration is measured as the time series between the onset and offset of the APA. During the body transportation phase, which includes the period between the leading heel contacting the anterior platform (Fig. 1c) and the back toe lifting off the posterior platform (Fig. 1d), we measured participants’ maximum lateral sway as well as the duration and velocity of their body transfer. The maximum lateral sway was measured as the maximum COP_NET-ML_ range of motion during the body transportation phase. The duration was calculated as the length of time during the body transportation phase and the velocity was calculated as the distance of the body transportation phase over the body transfer duration.

#### Clinical ratings of ASD severity

The ADI-R and ADOS-2 were used to examine ASD symptoms for each ASD participant and determine the extent to which postural control, APA, and stepping measures were associated with core symptoms of the disorder. The ADI-R [28] is a semi-structured parent/caregiver interview assessing current and past social interaction and communication behaviors characteristic of ASD, as well as the presence of restricted and repetitive behaviors. Higher scores reflect more severe abnormalities. The social, communication, and RRB algorithms were examined.

The ADOS-2 [29] is a semi-structured play-based assessment that uses developmentally appropriate social and play-based interactions to elicit behaviors commonly impaired in ASD, including language and communication, reciprocal social interaction, play, stereotyped behaviors, and restricted interests. Empirically derived social affect and restricted and repetitive behavior algorithm scores as well as severity scores ranging from 1 to 10 (1 being low severity and 10 being the highest severity) are calculated from raw ADOS-2 totals [38]. Severity scores were examined in relation to postural control and stepping outcomes. Severity scores were not available for individuals who completed Module 4 (*n* = 2).

### Statistical analyses

Data for each posture/stepping dependent variable were averaged across trials for each participant. We conducted a 3 (condition: neutral stance vs. Romberg one vs. circular sway) × 2 (COP direction: AP vs. ML) × 2 (group: ASD vs. TD) repeated measures ANCOVA to examine COP variability. In this model, condition and COP direction were the within-subject factors and group was the between-subject factor. Based on prior studies showing that height contributes significantly to individuals’ postural control and stability [39, 40] and its strong association with many of our primary dependent variables, height also was included as a covariate for all analyses. In order to assess MI and COP trajectory, we ran separate 3 (stance condition: neutral vs. Romberg one vs. circular sway) × 2 (group: ASD vs. TD) repeated measures ANCOVAs. The three conditions were the within-subject factor, and group was the between-subject factor. In cases where Mauchly’s test of sphericity was significant, results were interpreted using the Greenhouse-Geisser correction. In the case of significant interactions (*p* < 0.05), we ran post-hoc analyses using Bonferroni pairwise comparisons to correct for multiple comparisons. In order to examine anticipatory and body transportation phase processes during stepping, we compared anticipatory and body transfer variables between diagnostic groups using separate one-way ANCOVAs with height included as a covariate. Cohen’s d effect sizes also were calculated for group comparisons for all dependent variables [41]. An effect size of *d* = 0.2 was interpreted as small, *d* = 0.5 was considered a moderate effect size, and *d* > 0.8 was considered a large effect size.

To assess the relationships between postural control and stepping measures and symptom severity, we used Spearman correlations with ADOS-2 severity scores and restricted and repetitive behavior algorithm scores as well as ADI-R algorithm scores. Given that prior research has shown that postural control improves over childhood and into adolescence [20], we also examined the relationships between postural control, stepping measures, and age using Pearson correlations. Correlations with∣r∣ > 0.5 were interpreted as significant.

## Results

### Postural control during standing

The ASD and TD groups did not differ in COP variability as a function of stance condition (group × stance interaction: F(1.04, 34) = 0.02, *p* = 0.89; Table 2) or as a function of direction (group × direction interaction: F (1, 34) = 1.03, *p* = 0.32). Additionally, the ASD and TD groups did not differ in COP variability overall (F (1, 34) = 1.14, *p* = 0.29). There also were no significant effects of direction (F (1, 34) = 0.01, *p* = 0.87) or stance conditions on COP variability (F(1.04, 34) = 0.36, *p* = 0.56).

**Table 2:** Estimated means and effect sizes for postural control and step initiation measurements

	ASD (*n* = 17)	TD (*n* = 20)	Effect size	*p*
Neutral stance				
COP_ML_ (cm)	0.44 (0.06)	0.26 (0.05)	0.73	0.04*
COP_AP_ (cm)	0.61 (0.07)	0.47 (0.06)	0.50	0.15
MI (bit)	0.61 (0.04)	0.55 (0.04)	0.32	0.35
COP Length (cm)	34.65 (3.91)	24.11 (3.59)	0.65	0.06
Romberg 1				
COP_ML_ (cm)	0.77 (0.06)	0.62 (0.06)	0.58	0.10
COP_AP_ (cm)	0.83 (0.10)	0.58 (0.09)	0.62	0.08
MI (bit)	0.66 (0.02)	0.62 (0.02)	0.44	0.20
COP length (cm)	44.36 (4.01)	36.23 (3.68)	0.49	0.16
Circular sway				
COP_ML_ (cm)	7.36 (0.48)	6.89 (0.44)	0.24	0.49
COP_AP_ (cm)	3.80 (0.24)	4.03 (0.22)	0.24	0.49
MI (bit)	0.57 (0.04)	0.69 (0.03)	0.83	0.02*
COP length (cm)	454.84 (40.95)	351.81 (37.55)	0.61	0.08
Stepping				
Maximum APA (cm)	4.57 (0.49)	4.54 (0.48)	0.01	0.97
APA duration	0.49 (0.05)	0.43 (0.05)	0.29	0.41
Body transfer duration (second)	0.18 (0.01)	0.24 (0.01)	1.11	0.003**
Mean body transfer velocity (cm/second)	194.73 (14.65)	144.62 (13.43)	0.83	0.02*
Maximum ML (cm)	10.82 (0.80)	13.02 (0.77)	0.67	0.06

*Note. COP*_*ML*_ COP variability in the ML direction, *COP*_*AP*_ COP variability in the AP direction, *MI* mutual information, *COP Length* COP trajectory length, *Maximum ML* body transfer maximum lateral sway

Covariates appearing in the model are evaluated at the following value: Height = 155.130 cm. Effect size was calculated using Cohen’s d. Data are reported as estimated mean and standard error in parentheses

*p < 0.05 level; **p < 0.01

**Fig. 2:**
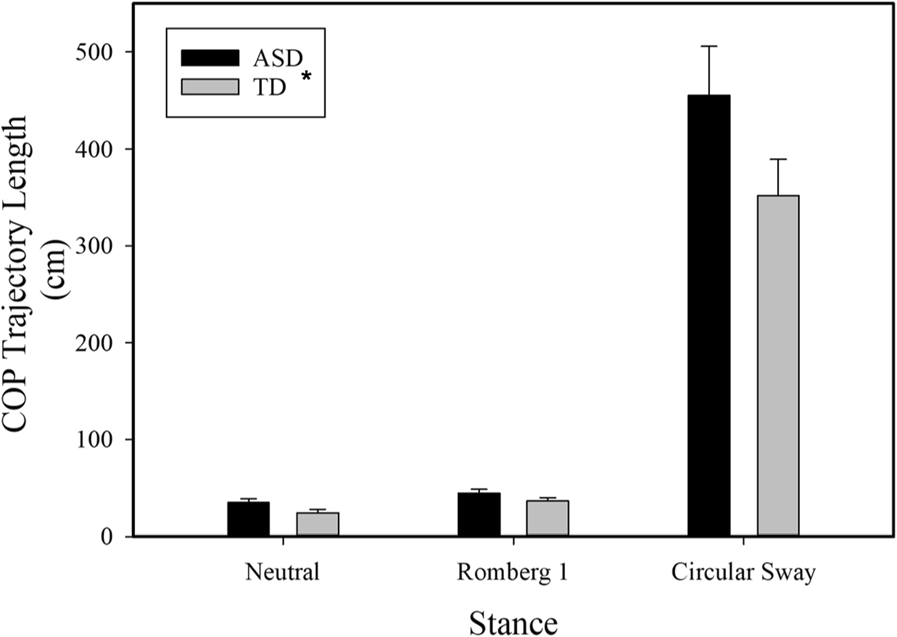
COP Trajectory Length*.* Covariates appearing in the model are evaluated at the following value: Height = 155.130 cm. There was a group main effect in which individuals with ASD showed significantly greater COP trajectory length than TD controls (*p* < 0.05). Data are presented as estimated mean and standard error bars

COP trajectory length did not vary between groups as a function of stance (stance × group interaction: F(1.01, 34.33) = 2.70, *p* = 0.11). Individuals with ASD showed greater COP trajectory length relative to TD controls (F (1, 34) = 4.17, *p* = 0.05; Table 2, Fig. 2). Across all participants, there was a significant effect of stance condition on COP trajectory length (F(1.01, 34.33) = 4.59, *p* = 0.04). Participants showed greater COP trajectory length during the circular sway condition compared to the Romberg one condition (F (1, 34) = 4.46, *p* = 0.04). There was no significant difference in trajectory length from the neutral stance to the Romberg one condition.

**Fig. 3:**
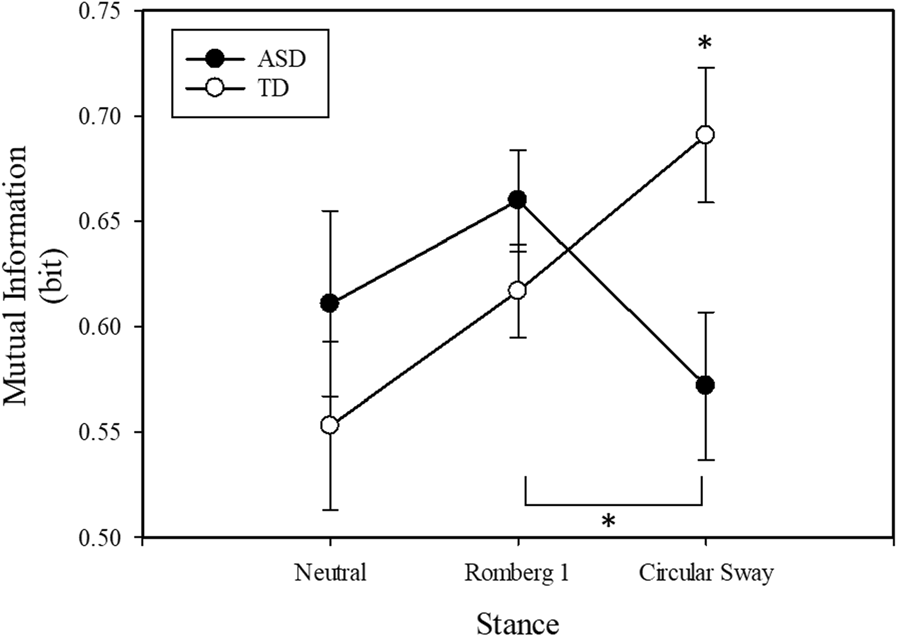
Mutual Information. Covariates appearing in the model are evaluated at the following value: Height = 155.130 cm. There was a significant group X stance interaction with individuals with ASD showing decreased MI during the circular sway condition relative to TD controls. Data are presented as estimated mean and standard error bars. *p < 0.05

Participants with ASD showed greater MI during the neutral stance and Romberg one conditions and reduced MI during the circular sway condition compared to TD controls (group × stance interaction: F(2, 68) = 5.03, *p* = 0.01; Table 2, Fig. 3). There was no main effect of stance condition on MI (F(2,68) = 1.47, *p* = 0.24).

### Postural control during stepping

**Fig. 4:**
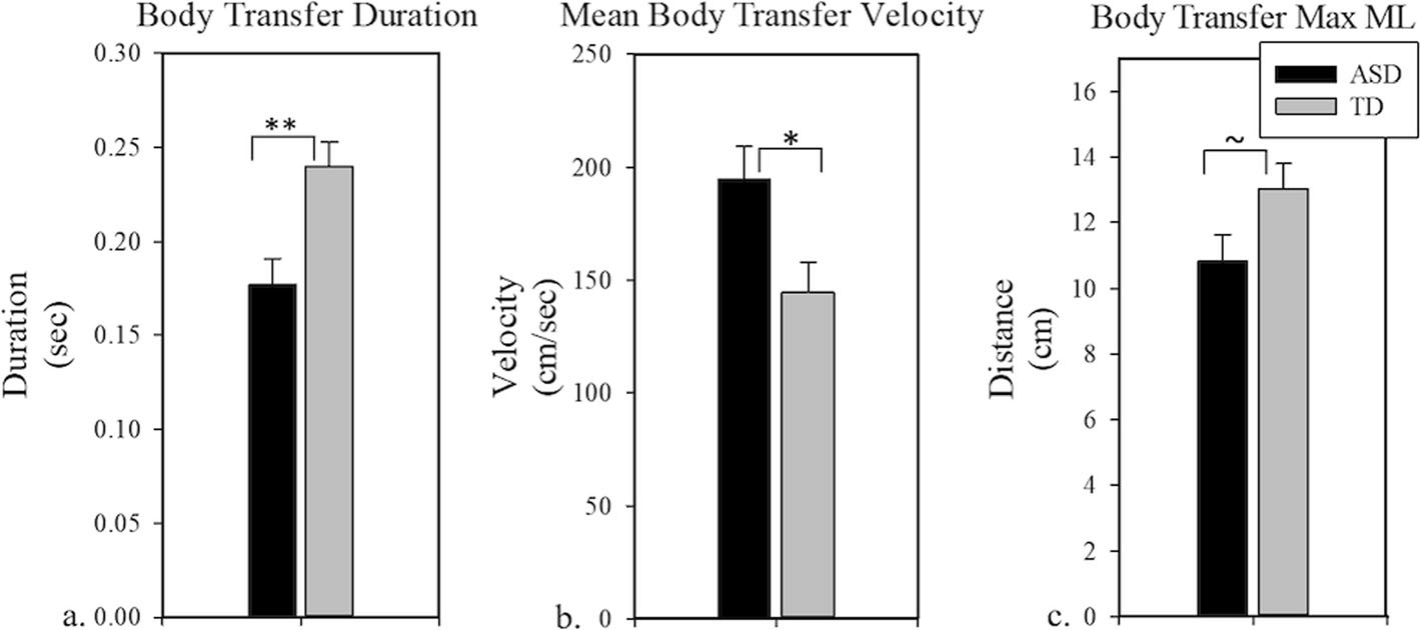
Body Transfer Phase. **a** TD individuals showed increased body transfer duration when stepping compared to individuals with ASD. **b** Individuals with ASD showed increased mean body transfer velocity when stepping compared to TD individuals. **c** Individuals with ASD showed decreased maximum lateral sway when stepping compared to TD individuals. Covariates appearing in the model are evaluated at the following value: Height = 155.130 cm. *p < 0.05; **p < 0.01; ~*p* = 0.063

During the anticipatory phase of the stepping task, individuals with ASD and controls did not differ on the maximum amplitude (F (1, 32) = 0.00, *p* = 0.97; Table 2) or the duration of their APAs (F (1, 32) = 0.71, *p* = 0.41). During the body transportation phase in which individuals shifted their COP from the back foot to their front foot, individuals with ASD showed greater body transfer velocity relative to controls (F (1, 34) = 5.97, *p* = 0.02; Fig. 4), as well as reduced body transfer duration (F (1, 34) = 10.49, *p* < 0.01; Fig. 4). They also showed reduced ML range of motion compared to controls, though this effect was marginal (F (1, 32) = 3.70, *p* = 0.06; Fig. 4).

### Demographic and clinical correlations

#### Associations between postural control outcomes

Intercorrelations between postural control and stepping variables were examined for each group separately. The correlations are summarized in Additional file 1 and Additional file 2. Briefly, neutral stance MI was positively associated with COP_ML_ variability (*r* = 0.62, *p* = 0.003) and COP_AP_ variability (*r* = 0.60, *p* = 0.005) during the Romberg one stance in TD controls but not individuals with ASD (COP_ML_ *r* = 0.01, *p* = 0.97; COP_AP_ *r* = − 0.03, *p* = 0.91). Additionally, neutral stance COP length showed a trend of negative association with ML range of motion during the stepping task (*r* = − 0.48, *p* = 0.05) in TD controls, while no association was seen in individuals with ASD (*r* = 0.02, *p* = 0.95). Circular sway COP_ML_ variability was positively associated with APA duration for TD controls (*r* = 0.63, *p* = 0.005) but not individuals with ASD (*r* = − 0.01, *p* = 0.96). In TD controls, there also was a positive association between circular sway MI and ML range of motion during the stepping task (*r* = 0.60, *p* = 0.009); this relationship was not significant for individuals with ASD (*r* = 0.1, *p* = 0.71).

Several postural control outcomes were correlated for individuals with ASD but not for TD controls. For individuals with ASD, neutral stance MI showed a trend of a negative association with neutral stance COP length (*r* = − 0.48, *p* = 0.05) but this pattern was not seen in TD controls (*r* = 0.02, *p* = 0.94). Individuals with ASD showed a positive association between neutral stance MI and circular sway MI (*r* = 0.54, *p* = 0.03), though no relationship was seen in TD controls (*r* = − 0.16, *p* = 0.51). Additionally, Romberg one MI was associated with decreased stepping velocity in individuals with ASD (*r* = − 0.59, *p* = 0.01), but not TD controls (*r* = − 0.02, *p* = 0.94).

#### Postural control outcomes and demographic features

For both the ASD and TD control groups, increased age was associated with decreased neutral stance COP_ML_ variability (ASD *r* = − 0.60, *p* = 0.01; TD *r* = − 0.62, *p* = 0.004), decreased neutral stance COP trajectory length (ASD *r* = − 0.71, *p* = 0.001; TD *r* = − 0.59, *p* = 0.01), decreased Romberg one COP trajectory length (ASD *r* = − 0.69, *p* = 0.002; TD *r* = − 0.57, *p* = 0.010), increased Romberg one MI (ASD *r* = 0.50, *p* = 0.04; TD *r* = 0.63, *p* = 0.003), increased circular sway MI (ASD *r* = 0.58, *p* = 0.01; TD *r* = 0.52, *p* = 0.02), and increased body transfer duration when stepping (ASD *r* = 0.59, *p* = 0.01; TD *r* = 0.73, *p* < 001). For both groups, increased height was associated with increased Romberg one MI (ASD *r* = 0.58, *p* = 0.01; TD *r* = 0.52, *p* = 0.02), circular sway MI (ASD *r* = 0.60, *p* = 0.01; TD *r* = 0.52, *p* = 0.02), and body transfer duration when stepping (ASD *r* = 0.65, *p* = 0.005; TD *r* = 0.75, *p* < 0.001). In TD controls, increased height was associated with increased lateral sway when stepping (*r* = 0.68, *p* = 0.002). For individuals with ASD, increased height also was associated with decreased neutral stance COP_ML_ variability (*r* = − 0.57, *p* = 0.02) and decreased COP trajectory length during neutral (− 0.68, *p* = 0.003) and Romberg one (*r* = − 0.69, *p* = 0.002) stance conditions as well as decreased body transfer velocity when stepping (*r* = − 0.67, *p* = 0.003). Correlation coefficients are provided in Table 3.

**Table 3:** Association between postural control and clinical/demographic features for individuals with ASD and TD control participants

	Age	FSIQ	Height	Weight	ADI-R Comm	ADOS-2 RRB
ASD						
Neutral COP_ML_	− 0.60*	− 0.12	− 0.57*	− 0.31	− 0.02	0.55^*^
Neutral MI	0.40	− 0.14	0.41	0.49	0.32	0.04
Neutral length	− 0.71**	− 0.17	− 0.68**	− 0.44	− 0.24	0.33
ROM1 MI	0.50*	− 0.15	0.58*	0.48	0.30	− 0.24
ROM1 length	− 0.69**	0.16	− 0.69**	− 0.39	− 0.15	0.25
Circle MI	0.58*	− 0.07	0.60*	0.57*	0.49	0.09
Circle length	− 0.43	− 0.04	− 0.35	− 0.38	− 0.34	0.20
Body transfer duration	0.59*	− 0.16	0.65**	0.64**	0.53^*^	− 0.04
Body transfer mean velocity	− 0.61*	0.09	− 0.67**	− 0.51*	− 0.50	0.18
Body transfer ML	0.06	0.12	0.06	− 0.11	− 0.07	− 0.55^*^
TD						
Neutral COP_ML_	− 0.62**	0.13	− 0.45	− 0.44	–	–
Neutral MI	− 0.09	− 0.06	−0.08	0.06	–	–
Neutral length	− 0.59**	< − 0.01	−0.47	− 0.44	–	–
ROM1 MI	0.63**	0.20	0.52*	0.39	–	–
ROM1 length	− 0.57**	0.15	−0.40	− 0.38	–	–
Circle MI	0.52*	− 0.02	0.52*	0.39	–	–
Circle length	0.06	< −0.01	−0.01	− 0.01	–	–
Body transfer duration	0.73**	0.22	0.75**	0.82**	–	–
Body transfer mean velocity	− 0.42	− 0.07	− 0.43	− 0.43	–	–
Body transfer ML	0.44	0.32	0.68**	0.48	–	–

*Note. Neutral* Neutral stance condition, *ROM1* Romberg one condition, *Circle* Circular sway condition, *COP*_*ML*_ COP SD in the ML direction, *COP*_*AP*_ COP SD in the AP direction, *MI* mutual information, *Body transfer ML* maximum lateral sway of body transportation phase

*p < 0.05 level; **p < 0.01 level

For individuals with ASD, greater neutral stance COP_ML_ variability (*r* = 0.55, *p* = 0.02) and decreased lateral sway when stepping (*r* = − 0.55, *p* = 0.02) were associated with more severe ADOS-2 algorithm ratings of restricted repetitive behaviors. Increased body transfer duration when stepping (*r* = − 0.53, *p* = 0.05) was associated with more severe clinical ratings of communication abnormalities based on the ADI-R. Decreased body transfer velocity when stepping (*r* = − 0.50, *p* = 0.07) also was marginally associated with more severe ADI-R ratings of communication abnormalities (Table 3).

## Discussion

This study examined postural control during both static and dynamic conditions in order to characterize reactive motor control processes made in response to sensory feedback, coordination of distinct joint processes, and feedforward postural adjustments in ASD. Five key findings are reported. First, individuals with ASD showed increased COP trajectory length across stance conditions compared to controls suggesting that they are less stable during standing. Second, individuals with ASD showed reduced MI during circular sway relative to controls suggesting a reduced ability to effectively coordinate distinct joint processes in order to maintain stability. Third, there were no differences between individuals with ASD and controls in the amplitude or duration of APAs suggesting that feedforward mechanisms involved in anticipatory adjustments prior to stepping are relatively unaffected in ASD. Fourth, during the body transportation phase of stepping, individuals with ASD showed reduced lateral sway, reduced body transfer durations, and greater body transfer velocities than TD controls suggesting that they are less stable when stepping. Last, greater neutral stance COP_ML_ variability and decreased lateral sway when stepping were associated with more severe restricted and repetitive behaviors in ASD suggesting that deficits of postural control may contribute to or reflect mechanisms overlapping with core ASD symptoms. Taken together, these results suggest that individuals with ASD show impairments involving multiple motor control mechanisms supporting postural stability and that these impairments may contribute to motor issues seen in everyday activities such as walking.

### Feedback guided reactive adjustments of postural stability

Maintaining postural control during static standing involves reactive motor adjustments guided by sensory feedback inputs including visual, proprioceptive, somatosensory, and vestibular information [11]. Our finding of increased COP trajectory length in ASD is consistent with prior work showing greater trajectory length and COP variability in ASD and suggests that feedback mechanisms supporting postural stability are compromised [5, 18]. These results indicate that sensory processing, or the integration of multiple sensory inputs, is aberrant in ASD and contributes to deficits in basic motor control. This hypothesis is consistent with prior studies of individuals with ASD demonstrating reduced integration of multiple sensory feedback processes during precision gripping [36, 42], over-reliance on dominant sensory inputs during gross motor behaviors and motor learning [43, 44], and disruptions of multisensory integration during postural control [3]. Therefore, reduced integration of multiple sensory feedback processes appears to disrupt multiple motor behaviors in ASD.

In contrast to our hypothesis and prior work, we did not find any differences in COP variability between individuals with ASD and controls. Given the significant difference between groups for COP trajectory length and the medium effect size difference between groups for COP_ML_ variability during static stances, the null findings reported here may simply reflect a lack of statistical power. Another possible explanation for this finding is that elevations in COP variability are more severe for individuals with ASD and comorbid intellectual disability (ID), as we only studied participants with average or above average IQs. Previous studies have shown that individuals with ASD and lower cognitive abilities show reduced postural stability relative to those with higher IQs [3, 45]. While we did not see a relationship between IQ and COP variability, this may reflect the restricted IQ range of our sample (> 70). We also found that increased age was associated with reduced COP variability in ASD. This finding suggests that postural stability may show a protracted course of development in individuals with ASD, but that it reaches similar levels as TD individuals during later childhood or adolescence.

### Coordination of distinct motor processes during postural control

We found that individuals with ASD showed reduced MI during circular sway suggesting an inability to coordinate distinct motor control processes during a task where fluid coordination is required. Healthy individuals show increased coordination of joint movements in order to maintain stability during more challenging postural conditions (e.g., feet heel to toe with one foot forward) [22]. The circular sway condition studied here involves the need to coordinate the timing of engagement of separate joint processes in order to support greater fluidity of non-linear movements. During static stances, including neutral or Romberg one stances, shared dependency of separate joints is less than that during circular sway in healthy individuals. Our findings of increased MI in ASD relative to controls during static stances is similar to findings of relative increases in shared dependency between joints during static standing seen in patients with neurodegenerative disorders [37]. These results also are consistent with previous findings that individuals with ASD show elevated MI during intentional sway along a single axis and suggest failures to decouple distinct control processes supporting postural adjustments in either the ML or AP directions [18]. When standing still or during a single direction sway, the increased MI seen in individuals with ASD may suggest a compensatory process in which they increase their body sway in multiple directions in order to decrease the likelihood of losing balance [18]. Alternatively, the decreased MI during the dynamic circular sway condition may reflect reduced adjustments used during the more complex dynamic movements which could be due to greater rigidity or reduced central coordination of distinct movement processes. Overall, individuals with ASD showed a reduced ability to flexibly modulate the amount of shared coordination between hip and ankle joints across distinct postural conditions.

We found that increased MI was associated with reduced COP trajectory length during the Romberg one and circular sway conditions, but not the neutral stance in TD controls. This suggests that during more challenging stance conditions, TD controls are able to increase coordination between adjustments in the AP and ML directions in order to decrease sway. While MI and COP trajectory length were not related during the neutral stance in TD controls, they were associated in ASD indicating that individuals with ASD utilize a similar strategy during the more basic neutral stance condition as demonstrated by TD controls during more challenging postural tasks. Together, these findings suggest that individuals with ASD may be compensating for failures to limit sway by coordinating movements in the AP and ML directions even during basic neutral stance conditions.

The postural control issues seen here in ASD may reflect alterations of cortical-cerebellar networks. The cerebellum plays a key role in the coordination of movements including the ability to make feedback guided reactive corrections to ongoing motor behaviors [46] as well as temporal and spatial coordination of distinct joint processes [47, 48]. Reduced coordination between hip and ankle joints during circular sway in ASD suggests cerebellar mediated deficits in the coordination of distinct postural control mechanisms [49]. This hypothesis is consistent with histopathological studies showing that cerebellar circuits involved in postural control develop atypically in ASD [50, 51] and functional magnetic resonance imaging (fMRI) studies documenting atypical functional connectivity between cerebellum and motor cortex during rest [52, 53] and gross motor behaviors [54].

### Feedforward and feedback motor control mechanisms supporting postural control during stepping

We did not find evidence of APA abnormalities consistent with feedforward control deficits during stepping in ASD. Our results suggest that abnormalities during stepping may not reflect the same deficits of anticipatory control seen in other planned motor movements or that not all individuals with ASD experience deficits in feedforward motor control. It also is possible that the lack of differences in APAs between individuals with ASD and TD controls is due to the self-timing nature of the task in which participants were asked to take a step when they were ready rather than requiring them to take a step immediately after receiving an auditory cue, which reduced the task difficulty but is more consistent with the self-paced nature of everyday postural actions. Further, our findings differ from a previous study in ASD showing reduced lateral sway during the first phase of gait initiation [5]. A difference in methods of step initiation measurement across studies may have contributed to the difference in results. Specifically, Fournier et al. (2010) examined gait initiation while participants stood with each foot on two adjacent force platforms and calculated the lateral sway by measuring the difference between the COP and the maximum center of mass while the current study measured step initiation while participants stepped from one force platform to an adjacent anterior force platform. Additionally, participants in the Fournier et al. (2010) study showed a significantly reduced IQ compared to the control group and relative to the present study which may have contributed to the discrepancy between studies.

Our results indicated that feedback control of stepping is disrupted in individuals with ASD. When stepping and moving from stationary to walking, momentum in the ML direction and a lateral shift towards the stance leg are required to maintain stability [5]. We found that participants with ASD showed decreased lateral sway suggesting that their movements are more rigid, or that they compensate for decreased postural stability by reducing the lateral amplitude of their sway [5]. Additionally, we found a reduction in body transfer duration during the body transportation phase in individuals with ASD which may suggest similar deficits in the AP direction as previously demonstrated in the ML direction [5]. Previous ASD studies have examined stepping using only one force platform; however, the present study involved participants stepping from one force platform to an adjacent force platform allowing us to measure important body weight transportation processes in the AP direction in addition to the ML direction. Overall, our findings suggest that feedforward mechanisms of stepping remain intact in this sample of individuals with ASD; however, deficits in feedback control during the body transportation phase results in greater instability when stepping.

ASD participants’ pattern of greater rigidity and decreased balance when stepping is similar to that seen in aging individuals and individuals with neurodegenerative disorders such as Parkinson’s Disease (PD) [55, 56]. Decreased lateral sway and reduced body transportation duration in ASD may reflect neural processes similar to those implicated in PD including dysfunction of basal ganglia circuits, consistent with prior structural and functional MRI studies of ASD (for review see Subramanian et al. 2017). Our findings also are consistent with a prior study showing that individuals with ASD had gait patterns similar to individuals with striatal dysfunction and resemble parkinsonian gait as evidenced by decreased stride length [23]. Additionally, in individuals with PD, stride kinematics are associated with putamen and nucleus accumbens volumes [57] suggesting that the atypicalities of postural control processes in ASD seen here may involve basal ganglia alterations.

### Associations between postural control deficits and ASD severity

We found that increased COP_ML_ variability during neutral stance and decreased ML sway during stepping each were associated with more severe clinically rated repetitive behaviors. These results are consistent with previous studies showing that reduced postural symmetry [26] and increased postural sway [25] in ASD are associated with more severe repetitive behaviors. Our findings suggest that shared neural mechanisms may be responsible for the development of both motor control impairments and restricted and repetitive behaviors in ASD, or that one of these deficits may cause the other. The basal ganglia has been implicated in repetitive behaviors in mouse models of ASD [58] and in clinical studies of ASD [59]. Specifically, MRI studies of individuals with ASD have shown an association between repetitive behaviors and striatal volumes [59–62]. The basal ganglia also plays a key role in learning and completing complex motor movements [63, 64] suggesting that alterations of basal ganglia development and its cortical targets may impact both basic motor control and more complex behavioral flexibility abilities in ASD. Functional neuroimaging studies are needed to clarify possible mechanisms linking basic postural control deficits and repetitive behavior issues in ASD.

### Limitations and future directions

This study presents new evidence for multiple distinct forms of postural control deficits in ASD. When interpreting these findings, multiple study limitations should be considered. First, this was a small sample study conducted across a relatively wide age range (6–19 years). Larger developmental studies across the lifespan are warranted to more clearly determine growth rates and patterns of distinct postural control processes in ASD. Second, we excluded participants with ID, though data suggest that postural control and goal-directed movements may be more severely impacted in individuals with comorbid ID [65]. Third, the inclusion of a TD control comparison group with no personal or family history of psychiatric or neurological disorders, or developmental disabilities may limit the generalizability of our findings. Another limitation is that our groups were not matched on height and weight. While both height and weight may be associated with postural control, the stronger relationship between weight and postural control primarily is seen in obese individuals [66, 67]. In the current study, the mean BMI across both groups was in the average range suggesting that weight did not have a significant impact on our findings (consistent with the smaller correlations with our posture and stepping dependent variables relative to height). Finally, comparisons of distinct motor control processes should be examined across different types of behaviors (e.g., upper limb, oculomotor) to determine the specificity of the pattern of motor deficits documented here to postural control systems.

## Conclusions

Overall, our findings identify deficits of joint coordination and sensory feedback processes during postural control in ASD. These findings, in the context of relatively intact feedforward mechanisms, provide new insights into discrete motor control and neurodevelopmental mechanisms associated with ASD. Additionally, this study highlights the need for future research examining discrete motor control deficits in early development and aging individuals with ASD and suggests targets for interventions that can be examined using precise and objective measurements. The relationships between these postural control deficits and core symptoms of ASD suggest that their study may provide important insights into neurobiological mechanisms contributing to both motor and core clinical issues in individuals with ASD.

## Supplementary information

**Additional file 1.** Correlation matrix of postural control and stepping variables for TD controls. This table provides the correlations between postural control and stepping dependent variables for TD controls.

**Additional file 2.** Correlation matrix of postural control and stepping variables for ASD individuals. This table provides the correlations between postural control and stepping dependent variables for ASD participants.

## Data Availability

Select data will be shared in the National Database for Autism Research (NDAR) according to agreements with NIH for the PI’s K23 award. We also would be open to share de-identified data based upon requests made to the first and last author of the paper.

## Abbreviations

ADI-R: Autism diagnostic interview-revised
ADOS-2: Autism Diagnostic Observation Schedule–Second Edition
AP: Anteroposterior
APA: Anticipatory postural adjustment
ASD: Autism spectrum disorder
BMI: Body mass index
COP: Center of pressure
ID: Intellectual disability
MI: Mutual information
ML: Mediolateral
PD: Parkinson’s disease
ROM1: Romberg one standing condition
SD: Standard deviation
TD: Typically developing
