# Supplementary material for: Postural control processes during standing and step initiation in autism spectrum disorder
Source: J Neurodev Disord. 2020 Jan 6;12:1. doi: 10.1186/s11689-019-9305-x (PMC6945692; doi:10.1186/s11689-019-9305-x)
Supplement: Supplementary file 1 — Additional file 1. Correlation matrix of postural control and stepping variables for TD controls. This table provides the correlations between postural control and stepping dependent variables for TD controls. [file 11689_2019_9305_MOESM1_ESM.docx]

Additional File 1

Correlation matrix of postural control and stepping variables for TD controls

|  |  | Neutral | | | | | | Romberg 1 | | | | | | | | Circular Sway | | | | | | | | Step | | | | | | | | | |  |
| --- | --- | --- | --- | --- | --- | --- | --- | --- | --- | --- | --- | --- | --- | --- | --- | --- | --- | --- | --- | --- | --- | --- | --- | --- | --- | --- | --- | --- | --- | --- | --- | --- | --- | --- |
|  |  | COP_ML_ | COP_AP_ | | MI | | Length | | COP_ML_ | | COP_AP_ | | MI | | Length | | COP_ML_ | | COP_AP_ | | MI | | Length | | APA | | APA Dur | | Dur | | Vel | | ML | |
| Neutral | COP_ML_ | 1.00 |  |  | |  | |  | |  | |  | |  | |  | |  | |  | |  | |  | |  | |  | |  | |  | |  |
|  | COP_AP_ | .89** | 1.00 |  | |  | |  | |  | |  | |  | |  | |  | |  | |  | |  | |  | |  | |  | |  | |  |
|  | MI | .40 | .29 | 1.00 | |  | |  | |  | |  | |  | |  | |  | |  | |  | |  | |  | |  | |  | |  | |  |
|  | Length | .86^**^ | .92^**^ | .02 | | 1.00 | |  | |  | |  | |  | |  | |  | |  | |  | |  | |  | |  | |  | |  | |  |
| Rom1 | COP_ML_ | .82^**^ | .66^**^ | .62^**^ | | .60^**^ | | 1.00 | |  | |  | |  | |  | |  | |  | |  | |  | |  | |  | |  | |  | |  |
|  | COP_AP_ | .84^**^ | .73^**^ | .60^**^ | | .56^*^ | | .75^**^ | | 1.00 | |  | |  | |  | |  | |  | |  | |  | |  | |  | |  | |  | |  |
|  | MI | -.50 | -.49 | .32 | | -.66^**^ | | -.14 | | -.22 | | 1.00 | |  | |  | |  | |  | |  | |  | |  | |  | |  | |  | |  |
|  | Length | .97^**^ | .88^**^ | .38 | | .82^**^ | | .79^**^ | | .90^**^ | | -.51^*^ | | 1.00 | |  | |  | |  | |  | |  | |  | |  | |  | |  | |  |
| Circular Sway | COP_ML_ | -.15 | .17 | -.26 | | .07 | | -.33 | | -.07 | | -.05 | | -.10 | | 1.00 | |  | |  | |  | |  | |  | |  | |  | |  | |  |
|  | COP_AP_ | -.41 | -.29 | -.30 | | -.31 | | -.37 | | -.22 | | .35 | | -.33 | | .39 | | 1.00 | |  | |  | |  | |  | |  | |  | |  | |  |
|  | MI | -.64^**^ | -.54^*^ | -.16 | | -.61^**^ | | -.64^**^ | | -.40 | | .52^*^ | | -.58^**^ | | .14 | | .50 | | 1.00 | |  | |  | |  | |  | |  | |  | |  |
|  | Length | .12 | .27 | -.30 | | .28 | | .06 | | .10 | | -.36 | | .15 | | .52^*^ | | .29 | | -.46 | | 1.00 | |  | |  | |  | |  | |  | |  |
| Step | APA | -.22 | -.23 | -.35 | | -.20 | | -.25 | | -.05 | | -.13 | | -.11 | | -.01 | | .18 | | .40 | | -.13 | | 1.00 | |  | |  | |  | |  | |  |
|  | APA Dur | .16 | .48 | -.28 | | .30 | | -.12 | | .19 | | -.25 | | .21 | | .63^**^ | | -.06 | | -.11 | | .41 | | .01 | | 1.00 | |  | |  | |  | |  |
|  | Dur | -.52^*^ | -.34 | -.26 | | -.44 | | -.66^**^ | | -.37 | | .31 | | -.47 | | .46 | | .15 | | .50 | | -.12 | | .00 | | .45 | | 1.00 | |  | |  | |  |
|  | Vel | .45 | .28 | .48 | | .32 | | .80^**^ | | .37 | | -.02 | | .39 | | -.30 | | -.18 | | -.43 | | .08 | | -.15 | | -.43 | | -.74^**^ | | 1.00 | |  | |  |
|  | ML | -.49 | -.40 | -.23 | | -.48^*^ | | -.44 | | -.21 | | .30 | | -.43 | | .33 | | .21 | | .60^**^ | | -.13 | | .60^**^ | | .17 | | .43 | | -.24 | | 1.00 | |  |

*Note.* Neut = Neutral stance condition; Rom1 = Romberg one condition; Circ sway = Circular sway condition; COP_ML_ = COP SD in the ML direction; COP_AP_ = COP SD in the AP direction; MI = mutual information; Len = COP trajectory length; APA = Stepping APA amplitude; APA Dur = Stepping APA duration; Dur = body transfer duration; Vel = mean body transfer velocity; ML = body transfer maximum lateral sway; *p<0.05 level; **p<0.01.
