# Supplementary material for: Postural control processes during standing and step initiation in autism spectrum disorder
Source: J Neurodev Disord. 2020 Jan 6;12:1. doi: 10.1186/s11689-019-9305-x (PMC6945692; doi:10.1186/s11689-019-9305-x)
Supplement: Supplementary file 2 — Additional file 2. Correlation matrix of postural control and stepping variables for ASD individuals. This table provides the correlations between postural control and stepping dependent variables for ASD participants. [file 11689_2019_9305_MOESM2_ESM.docx]

Additional File 2

Correlation matrix of postural control and stepping variables for ASD individuals

|  |  | | Neutral | | | | | | | | Rom1 | | | | | | | Circular Sway | | | | | | | | Step | | | | | | | |
| --- | --- | --- | --- | --- | --- | --- | --- | --- | --- | --- | --- | --- | --- | --- | --- | --- | --- | --- | --- | --- | --- | --- | --- | --- | --- | --- | --- | --- | --- | --- | --- | --- | --- |
|  |  | | COP_ML_ | | COP_AP_ | | MI | | Length | | COP_ML_ | | COP_AP_ | | MI | Length | | COP_ML_ | | COP_AP_ | | MI | | Length | | APA | | APA Dur | Dur | Vel | | ML | |
| Neutral | | COP_ML_ | | 1.00 | |  | |  | |  | |  | |  |  |  |  | |  | |  | |  | |  | |  | |  | |  | |  |
|  | | COP_AP_ | | .73^**^ | | 1.00 | |  | |  | |  | |  |  |  |  | |  | |  | |  | |  | |  | |  | |  | |  |
|  | | MI | | -.23 | | -.31 | | 1.00 | |  | |  | |  |  |  |  | |  | |  | |  | |  | |  | |  | |  | |  |
|  | | Length | | .89^**^ | | .82^**^ | | -.48 | | 1.00 | |  | |  |  |  |  | |  | |  | |  | |  | |  | |  | |  | |  |
| Rom1 | | COP_ML_ | | .51^*^ | | .29 | | .01 | | .42 | | 1.00 | |  |  |  |  | |  | |  | |  | |  | |  | |  | |  | |  |
|  | | COP_AP_ | | .80^**^ | | .57^*^ | | -.03 | | .76^**^ | | .71^**^ | | 1.00 |  |  |  | |  | |  | |  | |  | |  | |  | |  | |  |
|  | | MI | | -.49 | | -.12 | | .29 | | -.38 | | -0.21 | | -.18 | 1.00 |  |  | |  | |  | |  | |  | |  | |  | |  | |  |
|  | | Length | | .80^**^ | | .47 | | -.10 | | .68^**^ | | .88^**^ | | .86^**^ | -.50 | 1.00 |  | |  | |  | |  | |  | |  | |  | |  | |  |
| Circular  Sway | | COP_ML_ | | -.09 | | .01 | | -.32 | | .09 | | -0.22 | | -.28 | .00 | -.23 | 1.00 | |  | |  | |  | |  | |  | |  | |  | |  |
|  | | COP_AP_ | | -.43 | | -.28 | | -.33 | | -.26 | | -0.29 | | -0.42 | .34 | -.49 | .44 | | 1.00 | |  | |  | |  | |  | |  | |  | |  |
|  | | MI | | -.43 | | -.32 | | .54^*^ | | -.42 | | -0.30 | | -0.27 | .39 | -.45 | .37 | | .35 | | 1.00 | |  | |  | |  | |  | |  | |  |
|  | | Length | | .15 | | .18 | | -.66^**^ | | .32 | | 0.01 | | -0.13 | -.25 | .07 | .40 | | -.04 | | -.57^*^ | | 1.00 | |  | |  | |  | |  | |  |
| Step | | APA | | -.31 | | -.37 | | -.20 | | -.36 | | -0.02 | | -0.34 | .14 | -.18 | .07 | | .61^**^ | | -.16 | | .12 | | 1.00 | |  | |  | |  | |  |
|  | | APA Dur | | .36 | | .34 | | .08 | | .17 | | 0.36 | | 0.14 | -.24 | .39 | -.01 | | -.45 | | -.24 | | .36 | | -.14 | | 1.00 | |  | |  | |  |
|  | | Dur | | -.32 | | -.11 | | .44 | | -.41 | | -.52^*^ | | -0.34 | .48 | -.51^*^ | .09 | | -.04 | | .48 | | -.32 | | -.11 | | -.07 | | 1.00 | |  | |  |
|  | | Vel | | .53^*^ | | .23 | | -.23 | | .56^*^ | | .65^**^ | | .57^*^ | -.59^*^ | .72^**^ | .06 | | -.16 | | -.26 | | .20 | | -.08 | | .20 | | -.80^**^ | | 1.00 | |  |
|  | | ML | | -.08 | | .02 | | .10 | | .02 | | 0.01 | | 0.10 | .18 | -.03 | .02 | | .36 | | .10 | | -.30 | | .33 | | -.44 | | -.26 | | .22 | | 1.00 |

*Note.* Rom1 = Romberg one condition; COP_ML_ = COP SD in the ML direction; COP_AP_ = COP SD in the AP direction; MI = mutual information; Len = COP trajectory length; APA = Stepping APA amplitude; APA Dur = Stepping APA duration; Dur = Body transfer duration; Vel = Body transfer mean velocity; ML = body transfer maximum lateral sway; *p<0.05 level; **p<0.
